# Telemedicine Networks of EHAS Foundation in Latin America

**DOI:** 10.3389/fpubh.2014.00188

**Published:** 2014-10-15

**Authors:** Ignacio Prieto-Egido, Javier Simó-Reigadas, Leopoldo Liñán-Benítez, Víctor García-Giganto, Andrés Martínez-Fernández

**Affiliations:** ^1^ICT Department, Rey Juan Carlos University, Fuenlabrada, Spain; ^2^Rural Telecommunications Groups, Pontifical Catholic University of Peru, Lima, Peru; ^3^EHAS Foundation, Madrid, Spain

**Keywords:** telemedicine, e-health, tele-stethoscopy, telemicroscopy, rural areas, ict4d

## Abstract

Rural areas in developing countries are characterized by lack of resources, low population density, and scarcity of communications infrastructure. These circumstances make it difficult to provide appropriate health-care services. This paper explains research results achieved by Enlace Hispano Americano de Salud – Hispano American Health Link (EHAS) and how they have contributed to improve healthcare in isolated areas of developing countries through the use of information and communication technologies (ICT). As the first step, EHAS always collaborates with public health systems to identify its communication and information needs. Based on the analysis of needs, EHAS does research on appropriate technologies to provide communication in each context and on information systems suited to needs of health personnel. In parallel, EHAS has worked to provide applications that, making use of the communications services installed, could improve the health-care services in these remote areas. In this line, solutions to improve epidemiological surveillance or to provide telemedicine services (like a digital stethoscope or a tele-microscopy system) have been developed. EHAS has also performed several researches trying to ensure the sustainability of their solutions and has summarized them in a Management Framework for Sustainable e-Healthcare Provision. Finally, the effort to spread acquired knowledge has crystallized in a book that details all the technologies and procedures previously mentioned.

## Introduction

**Figure 1:**
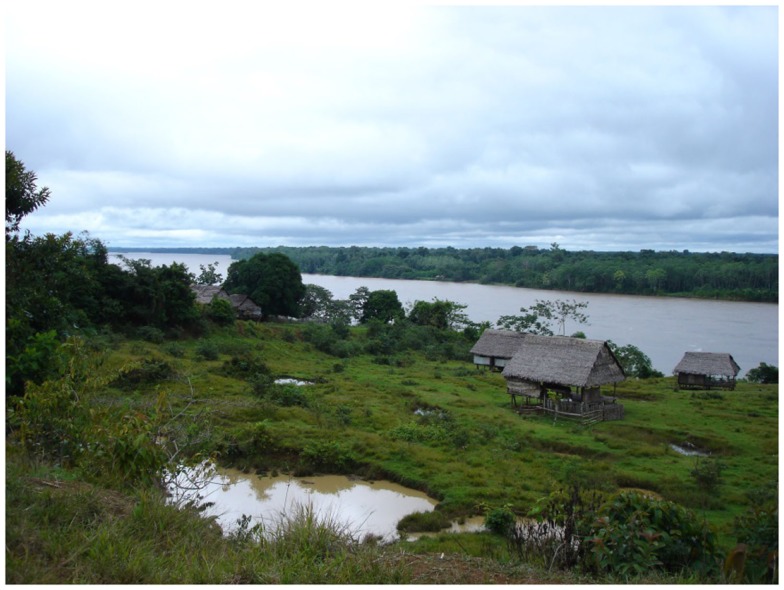
**Typical community in an isolated area without infrastructures**.

Healthcare in developing countries is not equally accessible to people living in urban or semi-urban areas and those living isolated rural areas. Rural areas, like the one shown in Figure 1, face important challenges such as the lack of resources (trained professionals, equipped establishments, power supply, etc.), high population dispersion, and the scarcity of communications infrastructure (roads, public transport, telecommunications, etc.). These circumstances make difficult to provide an appropriate health-care service to the population living in these areas. Therefore, it is precisely in this context where information and communication technologies (ICT) can make the difference. This is the main goal of Enlace Hispano-Americano de Salud (EHAS) Foundation; improving health-care services in isolated rural areas of developing countries, through an appropriate design and use of ICT. EHAS is a research and non-profit institution. Its board of directors is composed mainly by Spanish and Latin American universities (Technical University of Madrid, Rey Juan Carlos University, Pontifical Catholic University of Peru, and Cauca University in Colombia).

The work of EHAS began at the end of the 1990s by analyzing the communication and information needs in rural primary healthcare in developing countries. The primary health-care system in Latin America is usually composed by two types of facilities: health centers and health posts. Health posts are usually located in small communities and headed by health technicians that have received only a basic training. When a patient cannot be treated in the health post, he should be transferred to the referral health center, which is headed by physicians and have some infrastructure and equipment for diagnostic tests. In rural isolated areas, the trip from the health post to the health center can take 10 h in average and in urgent cases it can have a cost up to 2,000 USD. The last element of the primary health-care system is the hospital, where the patient can receive specialized medical attention. The analysis of information needs in this context started with a revision of existing studies on this matter (1–4), and continued by performing field researches in Peru and Nicaragua (5). The conclusions of that work showed that the main information needs in those areas where related to:

- Epidemiological surveillance: information arrives late due to the lack of communication infrastructures, it contains frequent errors because it is manually inserted several times in different locations, and it is not useful for taking correctives actions because it is not possible to send feedback on time.
- Correct diagnosis and treatment: it is impossible to the rural personnel to access medical information or discuss with other professionals, and the drug delivery system is inefficient due to the coordination difficulties. Furthermore, isolation and insufficient professional updating causes that personnel with higher qualification (physicians, obstetricians, and nurses) prefers to move toward urban areas.
- Emergencies management: it is difficult to coordinate resources (health staff, vehicles, fuel …) to transfer the patient, and it is hard to predict when the patient will arrive to the reference establishment (this would be very helpful to have the heath staff ready to the attention).
- Continuous medical education was mandatory for health staff, but its implementation was restricted by the high travel costs and the lack of communication or post services.

Another important fact is that voice communication is considered by different studies as the most important service in rural areas of developing countries (6). These information needs could be addressed by different telemedicine solutions, but there are several barriers to the deployment of new technologies in these scenarios: lack of infrastructure and electricity, low purchase power of the health establishments, and high maintenance costs due to the great distances and the scarcity of trained people.

## Communication Networks for Isolated Areas

Commercial communication solutions were not designed for these environments because they do not take into account its technical and economical constrictions. After the mentioned initial studies, EHAS started to do research on two lines: appropriate technologies to provide communication in this context and information systems suited to the health personnel needs. One of the first steps in this process was to identify the requirements that a solution must fulfill to be considered sustainable: be very robust to reduce travels for maintenance, have low operation costs, and low power consumption because they will be powered using solar energy.

**Figure 2:**
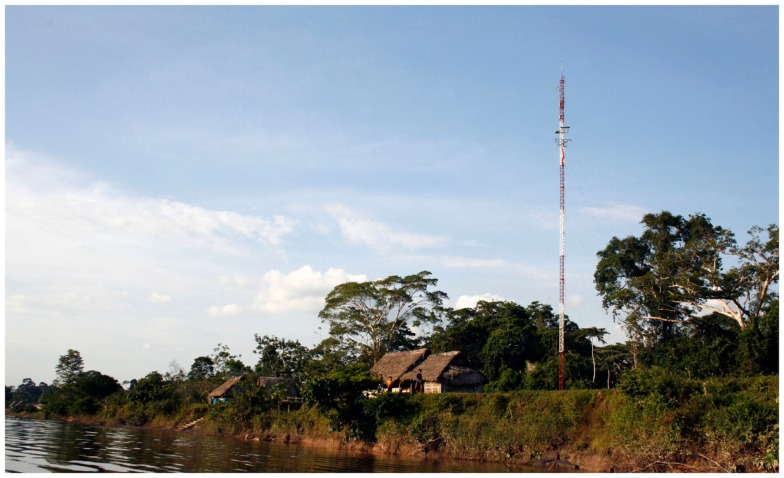
**Infrastructure of solar panels and tower installed in an isolated health posts**.

In order to face this communication challenge, wireless technologies appeared the most suitable ones. Most of the information needs could be addressed by asynchronous (off-line) systems with low-data transmission rates, such as email. Following this guidelines, two prototypes were designed focusing on the needs of rural staff. These prototypes were based on open technologies (hardware and software) in order to facilitate appropriation, and reduce acquisition, development, and maintenance costs. They were based on very high frequency (VHF) and high frequency (HF) radios to provide voice and low-data rate services over long-distance links. The prototypes used the sound card of a personal computer (PC) to modulate with the digital information (such as emails) an analog sound signal and send it through the audio channel provided by the VHF or HF radio. Using these prototypes, two pilots were deployed to communicate more than 75 isolated health posts in Colombia (7) and Peru (8), like the one shown in Figure 2.

**Figure 3:**
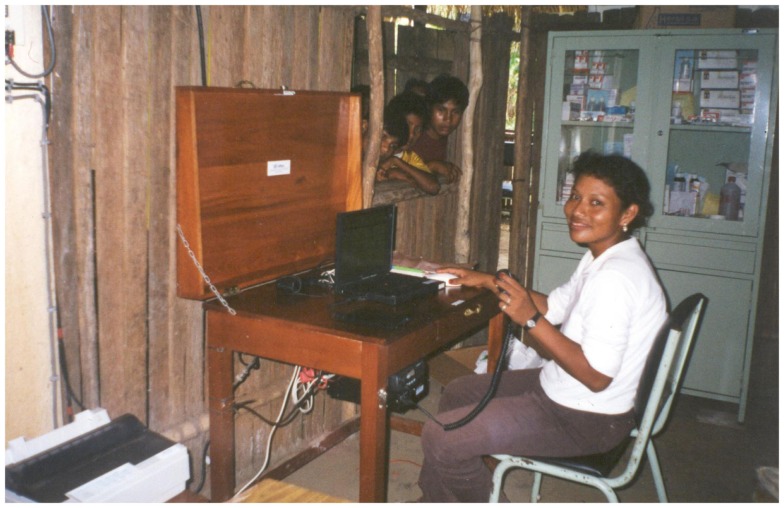
**Health technician using the VHF system to coordinate an emergency**.

The pilots served to measure the reliability of the technology and its impact on the health system (both in the staff and in the health-care). After 9 months of operation in 39 health posts of Peru, evaluation results showed that the mean consultation rate per facility was increased from 3 per month (95% CI 1.5–4.5) to 23 per month (95% CI 14.7–31.5). Moreover, there were 205 emergency transfers and the system was used in all cases to coordinate (as shown in Figure 3) and alert the referral center. Finally, the mean time required to evacuate a patient was reduced from 8.6 to 5.2 h (8). This field research in Peru was conducted with the help of the Rural Telecommunications Group of the Pontifical Catholic University of Peru.

**Figure 4:**
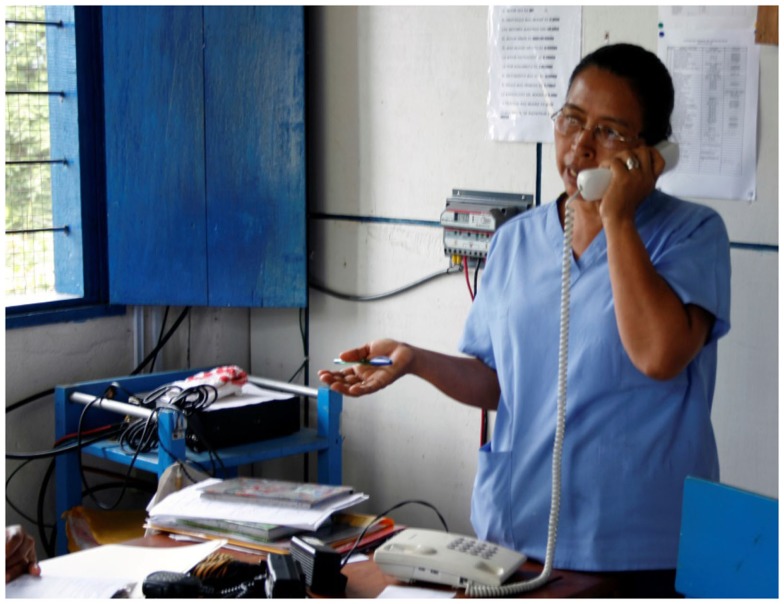
**Health technician using the VoIP system**.

Although the impact of these technologies proved to be very important, the bandwidth provided was still quite low (17 and 3 kbps with VHF and HF, respectively) and it was not possible to use them to transfer images or videos. At that time, some works (9, 10) had remarked the strategic role that IEEE 802.11 (the WiFi standard) could play for deploying low-cost networks in such scenarios. Besides, WiFi devices had spread and become very popular (due to its low cost) all over the world at that point, and EHAS decided to test if it was possible to use that technology to provide high transmission rates over long-distance links. The result to this question was positive (11), and some adjustments in the WiFi protocol (specifically in the ACKTimeout, CTSTimeout, SlotTime, and CWmin parameters) were proposed to improve significantly its performance over long distances. Learning from previous experiences like (12) and (13), this technology was used to deploy Wi-Fi for Long-Distances (WiLD) networks in Cuzco and Iquitos (14), both in Peru. Commercial embedded computers with a Linux Voyage distribution were used as WiFi transmission systems (15), and new antennas (suitable for WiFi frequencies) were installed in the towers previously used for VHF/HF systems. These networks were employed to provide three basic services: internal telephony, email, and Internet access. WiFi networks were not devised initially to support voice services, but voice over IP (VoIP) allows providing this kind of service like shown in Figure 4. Thanks to it, a telephony system was installed to allow health establishments to communicate among them without paying for calling time, and to call to the public telephone network using prepaid cards.

For the deployment of these pilots, it was not only necessary to develop the prototype but it was also required to do research on how to adapt the deployment to context conditions. The lack of electricity was solved by developing an autonomous wireless node for WiLD networks powered through solar energy (15) with 75 W panels and 12 V batteries. It was also necessary to design the system considering environmental characteristics such as tree height, humidity, and thunderstorms in the Amazon jungle, or mountain altitude and ground conductivity in the Andean region. Approximately, the throughput in these networks was limited to 3 Mbps, so some research on how to optimize resources and maintain the quality of main services (QoS) was also performed (16).

In the last years, EHAS has continued its quest for innovative communication solutions for rural isolated areas, studding the latest versions of 802.11 standard and new technologies such as IEEE 802.16 (known as Wimax) (17). This work has served to improve existing networks and use them to provide services requiring more network resources, like the telemedicine services that will be explained in the next section.

Nowadays, communication systems are converging toward IP and mobile technologies, and EHAS is working to bridge another important aspect of the digital divide: the access to mobile services in rural isolated areas of developing countries. Mobile telephony and Internet access bring the opportunity to break isolation by reducing barriers to knowledge acquisition and participation, and by allowing these communities to access new economic opportunities (18, 19). Different studies have shown that the contribution of mobile telephony to economic development is greater in less developed economies, and that this technology impacts directly on the reduction of poverty levels. Specifically, a study of the World Bank proved that a 10% increase in bandwidth penetration can cause a 1.4% rise of the gross domestic product (GDP) (20). However, isolated areas are not profitable for mobile operators due to the low density population and the high cost of infrastructures. To face this problem, EHAS is participating (together with 10 more partners) in a research project known as TUCAN3G that is being funded by the European Commission through the FP7 (21). Its objective is to prove that it is possible to use low-cost 3G station bases designed for indoor scenarios (known as femtocells) to provide mobile voice and data services (3G) in isolated areas of developing countries, using a heterogeneous WiFi/WiMAX-VSAT network as backhaul. The research is developing solutions to adapt and integrate the different technologies used, and is also trying to develop a sustainable business model in order to involve mobile operators in the solution. The participation of operators is required because they have the licenses to operate in the mobile bands. If this solution proves feasible, it could help to cover communication needs of the primary health system in rural isolates areas.

## Telemedicine Services

In parallel with the work on communication technologies explained in the previous section, EHAS has done research to develop applications that, making use of the communications networks installed, could improve both the health information system and the health-care services in these remote areas. The first approaches in this line focused on increasing the efficiency of the epidemiological surveillance system and allowing distance training in remote areas (22) using voice and low-rate data systems based on VHF.

The need to improve health information systems is related with the existence of prevalent diseases such as dengue, malaria, infectious respiratory diseases, or diarrhea diseases. Having updated and accurate information of a disease situation is crucial to prevent or promptly face an epidemic. When EHAS started its work, most of the existing research was oriented to the analysis of data, although the main problems in the rural areas were related with the collection process. Therefore, EHAS contribution was to develop an appropriate solution to collect this information using low-cost and open-source technologies designed for these specific contexts. The Telematics Department of the University of Cauca developed a system for collecting, sending, processing, visualization, and feedback of epidemiologic information. This system was designed for scaling at national level, but it was previously tested in a pilot area, where it solved the problem of subregister, increasing the volume of data collection a 15%. A similar approach was used to design a new distance training system, given that existing solutions were based on video transmission (which requires a high a bandwidth that was not available) or required permanent connection to Internet (which was hard to guarantee in remote locations). A distance training system synchronized through email messages was developed by the Technical University of Madrid and the Carlos III University of Madrid. Both systems were based on a combination of email and XML (eXtensible Markup Language) technologies and were designed to work over slow, unreliable, and asynchronous connections. These solutions were used to offer remote courses about “child diarrhea,” “nursery attention in the primary health-care pre-emergency services,” “epidemiologic surveillance,” and “health education and disease prevention, grass-root level oriented” in 52 health establishments of Dominican Republic, Cusco (Peru), Cauca (Colombia), and Guantanamo (Cuba).

The research on health information systems has continued since then and nowadays is based on the use of open-source tools such as OpenMRS (23) or DHIS2 (24). The advantages of using these tools in concrete countries, such as Peru, Paraguay, Mexico, or Colombia have been in deep studies, and currently DHIS2 is being deployed in some regions of Colombia (25). When the communications technologies started to provide high bandwidth networks (taking advantage of WiLD solutions) EHAS started to work on ways to improve the diagnosis capabilities of the rural establishments. The basic idea was to allow doctors to remotely support health technicians using real-time communications tools based on voice and video. In these scenarios, a remote consultation has great advantages:

- The patients feel safer knowing that some doctor is helping with their diagnosis. These systems increase patient willingness to attend health posts, in regions where reluctance to modern medicine is still very important.
- The health technicians can improve their training and provide a better service to their patients, which make them feel more confident.
- A remote diagnosis allows plenty of diseases to be treated in the health post, avoiding transferring the patient, and saving costs to the patient or to the health system.

**Figure 5:**
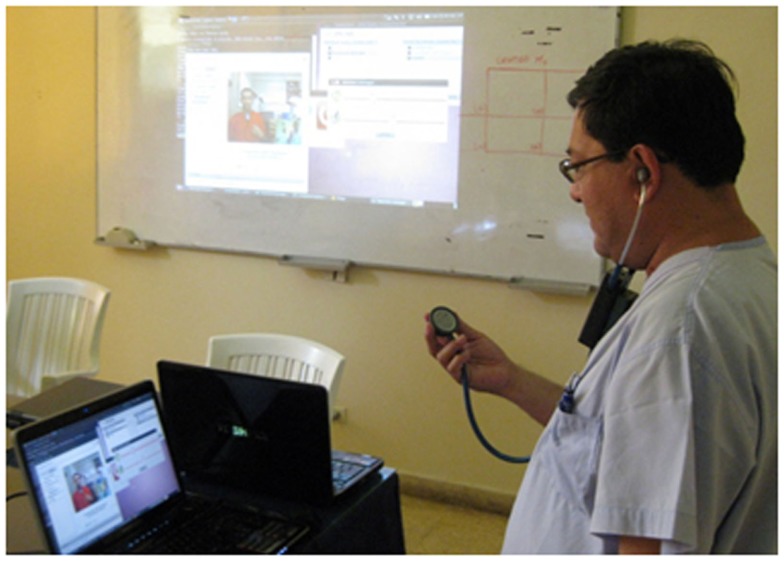
**Doctor using the digital stethoscope developed by EHAS Foundation**.

One of the first works in this line was a digital stethoscope (26) aimed to diagnose infectious respiratory diseases, which are the main infant mortality cause in rural areas of developing countries. When this digital stethoscope was developed there was no other device with similar performance, and nowadays there are solutions that send the sound in real time but do not offer a video of the patient. This digital stethoscope allows a doctor to support a health technician in the diagnosis process of respiratory or cardiac pathologies. It sends the audio and video in real time, so the remote doctor is able to know the position of the stethoscope associated with each sound, to ask the technician to change this position and to give instructions to the patient. This information is crucial for the doctor to make a remote diagnosis. The stethoscope also allows recording the sounds in order to send them in a file to ask for a second opinion. The information to build the digital stethoscope is shared under GPLv3 license (for free and open-source software). Its design is simple in order to make it possible to produce it in universities or small companies of developing countries. But at the same time, the design is robust enough to be used in areas with extreme climate conditions (high temperatures, high humidity rate, etc.). Moreover, the required components are low cost (approximately 200$) in order to avoid that the cost become a barrier for its use. All this characteristics has been thought to develop a suitable digital stethoscope shown in Figure 5 that can be used in rural areas of developing countries.

**Figure 6:**
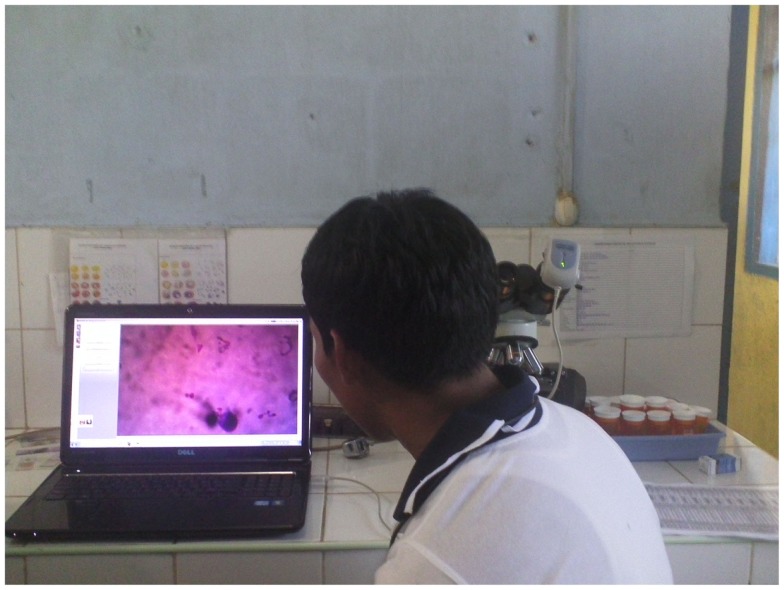
**Tele-microscopy system in a remote health establishment**.

A microscopy would be a very complementary tool for the digital stethoscope because “Pneumonia, diarrhea, and malaria together killed roughly 2.2 million children under age five in 2012” (27). However, health technicians do not have the knowledge to diagnose using the microscopy. To face this problem, EHAS is currently working in two lines in a project funded by the Spanish Agency for International Development Cooperation (AECID). First, EHAS is designing and evaluating very simple protocols to prepare microscopy samples with the resources available in remote health posts. Secondly, EHAS is developing a low-cost tele-microscopy system based on on-board computers (28) shown in Figure 6. The idea is to teach health technicians to prepare the microscopy samples and provide them with a tele-microscopy system. In this way, they would be able to send the microscopy images in real time to a specialist in order to get a diagnosis. Other two prevalent diseases in developing countries are included in this research: tuberculosis and cervical cancer. It is important to emphasize that both the protocols and the tele-microscopy system are being evaluated by specialist in order to guarantee that this solution provides a sensibility and specificity equivalent to a traditional diagnose.

**Figure 7:**
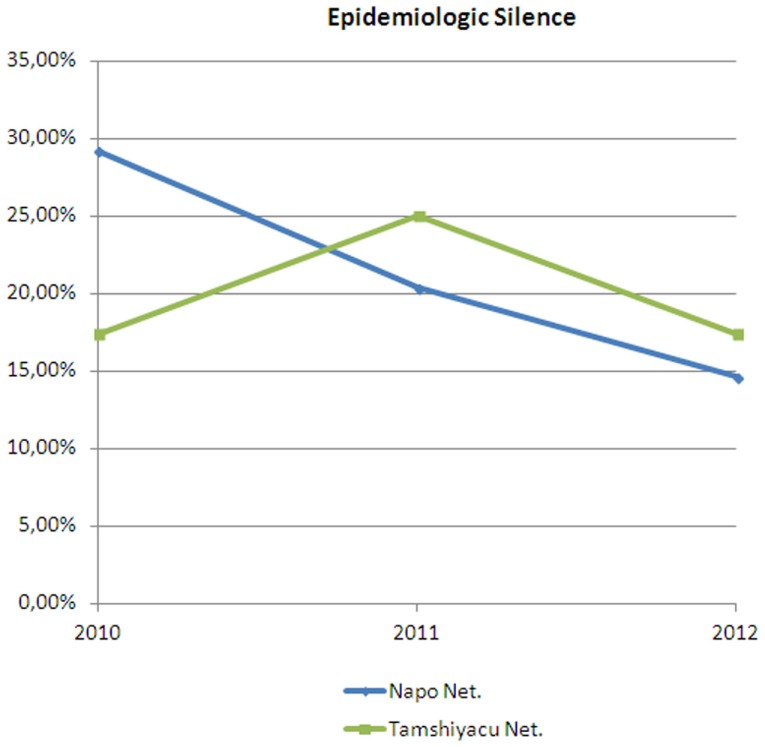
**Evolution of the percentage of epidemiologic reports lost before reaching the epidemiologic department**.

**Figure 8:**
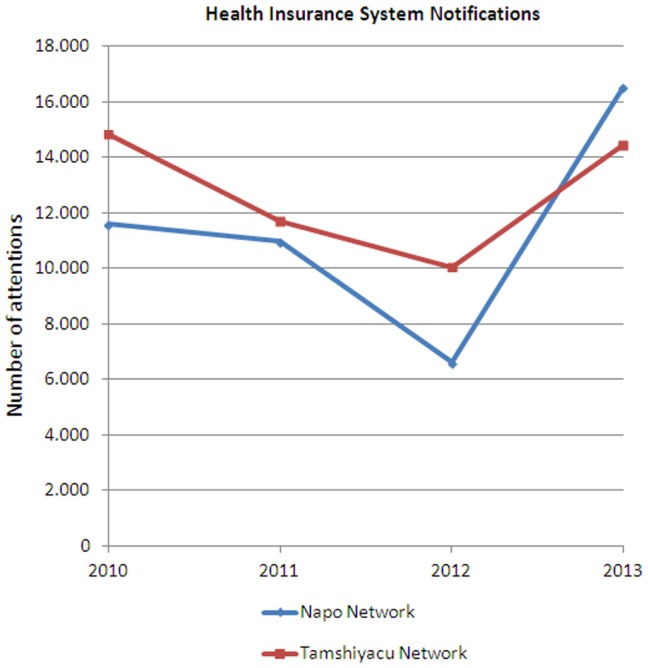
**Evolution of the number of attentions reported to the Health Insurance System of Peru**.

These telemedicine services are being used in the WILD network deployed in Iquitos, which is known as the Napo Network and that communicates 11 health posts with its reference health center and with the regional hospital. The project to deploy these services in this network started in 2010 by improving the communication and the information protocols. However, the telemedicine systems were not installed until 2013. The use of the networks is nowadays very intensive, the evaluation on the use of this equipment is still being performed, but the mean of VoIP call per health establishment is 2,000 calls per month (29). To evaluate the impact of these initiatives some indicators of the Napo Network are being compared with a control group. The selected control groups are the health establishments of the Tamshiyacu health network, that shows a high Pearson coefficient (*r* = 0,818), and has a similar demographic and isolation conditions as the Napo Network. The impact on health indicators is hard to measure and is still being evaluated, but the impact on processes has already been analyzed. Figure 7 shows the impact on epidemiologic silence (the percentage of health reports sent by the health establishment that get lost and do not reach the Epidemiologic Department of the Regional Government), showing a clear reduction in the Napo Network since 2010. The project also had an important influence on the exchange on information about the number of attentions that were notified to the Health Insurance System (SIS) of Peru (Figure 8). The number of notifications grows a 50%, and thanks to that the income of the health network (coming from the national Health Insurance System) increased an 80%.

**Figure 9:**
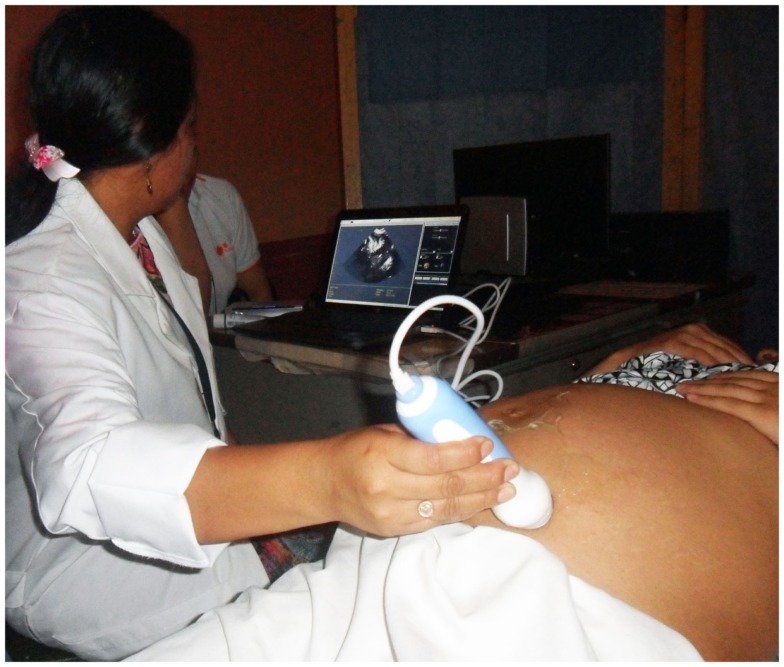
**Nurse performing a pre-natal check**.

Enlace Hispano-Americano de Salud is now performing a research project to reduce maternal mortality in rural areas of developing countries through the use of portable ultrasound scans (like the one shown in Figure 9) and blood tests. These tools are basic for pre-natal care, but they are unavailable in many rural areas of developing countries due to the lack of electricity and suitable technologies. EHAS is evaluating an innovative and cost-effective technology (a backpack containing a small solar panel and the required equipment for blood testing and ultrasound spot screening) to improve the access to and the quality of pre-natal care in these rural areas. Nurses are trained to locally use these tools and perform a basic check-up oriented to detect the most common obstetric complications. The information is stored in the computer offline and synchronized with a web platform when Internet connection is available. In this way, the information is later supervised by a specialist that confirms the diagnosis and improves the nurses training. These tests could detect 80% of obstetric complications on time, with an average cost of about US$ 25.00 per pregnant woman (including two ultrasounds, two blood, and two urine analysis). The aim is to convert an obstetric emergency (that means expensive transfers and attentions in many cases already useless) into a reference to an appropriate health center with a routine transfer 1 or 2 weeks before the labor. The first results obtained (with a sample of 1000 women) are very promising: the health mortality has been reduced to 0 and the newborn mortality has decreased a 50% (30).

## Sustainability

**Figure 10:**
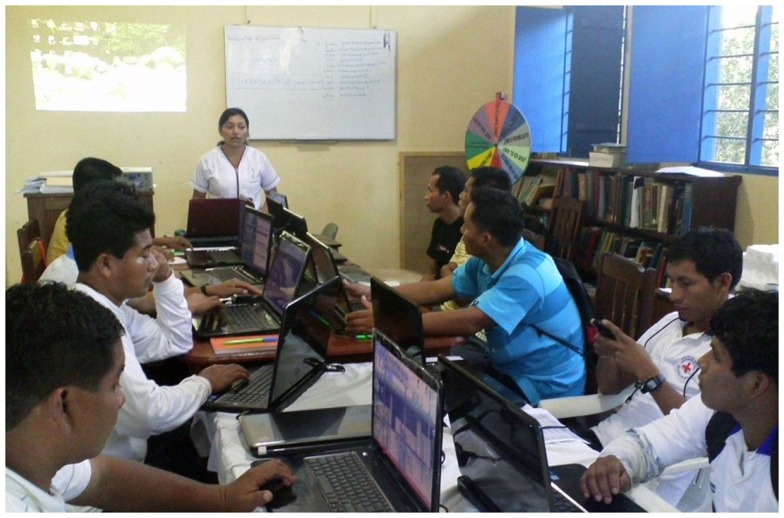
**Health technicians being trained in the use of health information systems**.

The technical feasibility of a solution could be proved in a laboratory test bed; however, developing interventions are very complex and achieving sustainability is not a mere technological issue. That is why EHAS Foundation always includes a pilot deployment in its research projects, as a way to consider other factors involved in sustainability, such as economic, financial, institutional, educational, and social and cultural aspects (31). In this process, local universities have always played an important role as local partners due to several reasons:

- They already have a technical background, so they are a suitable actor to lead the technology appropriation process. They can also perform the pilot deployment and guarantee the maintenance of the systems in the first stages of the pilot.
- They also have an educational background, very important to train the health staff in the use and maintenance of the new systems (Figure 10), to train maintenance experts, and to transfer the knowledge to other actors in the country.
- They can help to understand the cultural and institutional aspects of each local context, and to spread the achieved results among local and national institutions.

Enlace Hispano-Americano de Salud has based on most of its solutions in open-source software in order to facilitate technology transfer and to encourage other institutions to replicate and improve the proposed solutions. The VHF modem, the VoIP server, the health information systems, the digital stethoscope, and the tele-microscopy system previously explained are examples of this, but it also includes software to manage the networks (32), which is a very important issue in the network maintenance.

The economical factor is also studied in the pilots, proving that the net economic effect of the telemedicine program over a 4-year period was clearly positive, and that the additional operational costs introduced by the telemedicine system were lower than the savings produced for the health-care network (33). These savings were related to the reduction in patient referrals and the cost associated to them. Prior to the availability of the EHAS telemedicine system, there was a mean of 11.1 urgent patient referrals per year from the HPs and 14.0 referrals per year from the HCs. After the implementation of telemedicine, patient referrals fell to 2.5 (With a *P* value of 0.03) per year from the HPs and to 8.4 per year from the HCs (with a *P* value of 0.17).

In order to ensure sustainability and bring together all the experience related with sustainability, a Management Framework for Sustainable e-Healthcare Provision was developed (34). This framework considered human resources, logistics, and budget guaranteed for operation and maintenance of the network, and it was applied in the Napo Network. The network was transferred to the Regional Government, who is now responsible of the whole network and guarantees its continuity. This strategy implies an organizational change (definition of new roles, annual budget approval, etc.) that is also described in the framework.

Finally, the effort to spread the acquired knowledge has crystallized in several scientific papers and a book (35) that details all the technologies and procedures previously mentioned. This book aims to spread the use of ICT for health in rural areas of developing countries.

## Future Works

Until now, the challenge was to find communication technologies to connect isolated health establishments and to provide services such as remote training, support to diagnose, or information exchange. Nevertheless, nowadays the mobile systems have reached a high penetration even in the rural areas, and telemedicine solutions are expected to converge with mobile networks. Therefore, future research should make compatible traditional medical instruments (stethoscope, microscope, ultrasound scanner, etc.) with mobile operation systems such as android or iOS. In this way, developing countries could take advantage of the products developed in the industrialized areas, something that until now has not been possible. This is, especially, relevant in countries with a middle human development index, which nowadays have resources to hire medical specialists but where it is hard to find specialist willing to work in rural areas. In this context, telepresence is revealing itself as a promising alternative. In a complementary way, research on solutions to provide an automatic diagnose will provide useful screening tools and will help to promptly detect and treat the most serious cases. Strategic alliances to combine different technologies are other key point where research is needed. Remote consultation systems could learn from initiatives like “Water First – Health Follows” of the e-Health-Point project, where prevention (access to drinkable water) is combined in the same monthly fee with medical attention (in case prevention fails). Finally, an important effort is required to include medical teleconsultation in developing countries legislations as part of the portfolio of services in order to make refundable this type of attentions. This is an important step to transform an isolated set of pilot projects into a part of national health-care systems.

## Conclusion

The research line of EHAS Foundation has focused during more than 15 years on designing, developing, and evaluating ICT applications to face health problems in rural areas of developing countries. It started developing communication solutions for areas that were completely isolated, and the evolution of the technology allowed EHAS to drift toward more ambitious applications such as telemedicine services. Some of the networks here described have been working for more than 7 years since they were transferred to local actors, what proves that the sustainability plans developed are reaching their objective. The tele-stethoscopy and tele-microscopy systems have proved their sensibility and specificity through clinical trials (still in publishing phase), and several pilots are being evaluated to prove that it is possible and efficient to provide telemedicine services in remote areas. Results show that the impact is positive for the patients (that receive an improved healthcare), for health personnel (that has more resources, more opportunities and more confidence), and the whole health system (that increases available information and the health networks income). These results have contributed to spread telemedicine opportunities and in this line other telemedicine networks have been deployed in Peru, Ecuador, and Colombia. The next challenge is to find mobile technologies to make more accessible the ICT solutions, a field where EHAS is already working. These research results can be useful to all kind of actors (national public administrations, multilateral institutions, industry, academy, civil society, etc.) in order to promote really relevant and sustainable solutions in telemedicine for rural regions of developing countries.

## Summary of Projects

This section will provide a summary of the projects described in this paper, showing the most relevant figures of each one (Table 1).

**Table 1:** **Summary of EHAS projects**.

Project	Focused on	Patients/system	Cost/patient	Started	Ended
VHF networks	Synch. consultation on respiratory infections and diarrheal diseases and coordination of urgent transfers	5,000	US $2	2001	2012
	Asynch. coordination, tele-training and epidemiological reports	
Napo networks (WiLD)	Synch. consultations on obstetrics, pediatrics, and dermatology, and coordination of urgent transfers	8,500	US $4	2007	No
	Asynch. management of medicines stock and epidemiological reports	
Tele-stethoscopy	Synch. consultation on cardio-respiratory diseases	750	US $1	2012	No
Tele-microscopy	Synch. consultation on malaria, tuberculosis, parasitic infections, and cervical cancer	600	US $1	2013	No
Healthy pregnancy	Rural maternal and neonatal mortality reduction	400	US $25	2012	No

### Project I

EHAS – VHF Networks (7, 8, 22, 33). This Project was executed in Alto Amazonas (in 56 health establishments) and Datem del Marañon (in 17 health establishments), which are both provinces of the Loreto Department in Peru, and also in the districts of Silvia and Jambalo (in 21 more health establishments), which belong to the Cauca Department in Colombia. The funds were provided by the Andean Health Organization (ORAS) and the Spanish AECID. This project deployed communication systems for voice and data (for email access only) that were based on radio VHF links (using TCP/IP over AX.25). The first 39 Peruvian systems were installed in 2001 and the last 17 ones were installed in 2006, and all of them have been operating until 2012, date on which most of them were upgraded with WiFi over long-distance (WiLD) technology. The systems in Colombian were installed in 2004 and have been operating until 2010. Voice communications were used to query synchronous clinical questions (mostly related to respiratory infections and diarrheal diseases) and to coordinate urgent transfers. The data system was used asynchronously to coordinate activities, as a tool for tele-training and also to send epidemiological reports. The average cost per installation was US $5,500 per establishment (10% of annual maintenance included), which included a computer and a printer, a radio transceiver, towers and antennas for communication, a system for protecting against lightning, and a solar power supply solution. These systems have been operating an average of 7 years and have been used to attend more than 5,000 patients each, what implies an average cost of US $2 per patient (taking into account CAPEX and OPEX). This project had a high impact in the reduction of travel of health staff and in the coordination of urgent transfers, as has been stated previously.

### Project II

EHAS – Napo Network (11, 14, 15, 21, 29, 34). Project carried out in the basin of the Napo River, in the Maynas province, in Loreto, Peru. The Project deployed a telecommunications network with WiLD technology in order to connect 15 establishments of health along 450 km of river. The Project was initially funded by the ORAS in 2007, and was completed with funding from the Madrid City Council (Spain) in the year 2009 (the network is still operating nowadays). The initial equipment was composed by a VoIP telephone and by a computer with permanent access to the Internet and a videoconference software. The system has been used to perform synchronous consultations between the health technician and his reference doctor, and also between the doctor and the specialists of the regional hospital, particularly in pathologies related to obstetrics, pediatrics, and dermatology. The Internet connection has been intensely used to control the stock of medicines and send epidemiological reports. Video conferencing has been used for remote training of technicians, which reduces the number of trips, and therefore, the travel expenditures of the health system. The average cost per installation exceeded US $20,000 due to the need for high towers to secure the line of sight between antennas. Assuming equal maintenance costs than in the previous project, and taking into account that each establishment has attended more than 8,500 patients, the average cost per patient is US $4, without considering the effect of remote training and the improvement on information management. This project has a high impact in the reduction of urgent transfers, the reduction of epidemiological silence areas, and the maintenance of medicines stock.

### Project III

EHAS – Tele-stethoscopy (26). Fifteen tele-stethoscopes were installed in the network of the Napo River in order to provide remote cardio-respiratory auscultation services. This project was funded by the Madrid City Council in 2011 and continues operating nowadays. The system allows reference doctors to remotely monitor cases of acute respiratory infections. To a lesser extent, the system is being used so that general practitioners can receive a second opinion from the specialists in cardiology at Iquitos Regional Hospital. The cost of each system is US $600 per point (including installation costs) and has been used with more than 750 patients by establishment, which means a cost of less than a dollar per patient. Its impact on the reduction of morbidity and mortality has not been scientifically evaluated due to the lack of resources to study a randomized control group in such a remote area.

### Project IV

EHAS – Tele-microscopy (28). Fifteen tele-microscopy systems have been installed the last year in the Napo network, and are used to make remote diagnosis of malaria, tuberculosis, parasitic infections, and cervical cancer. This project has been funded by the Spanish AECID. The equipment and installation costs do not exceed US $500 and the estimated average usage is two daily cases by establishment.

### Project V

Healthy Pregnancy (30). The pilot project was performed in Alta Verapaz, Guatemala, between 2012 and 2013. It was funded by the Polytechnic University of Madrid, and it has equipped three nursing brigades with a portable ultrasound kit, a folding solar panel (for powering), and a system for performing blood tests using dried blood. The system is used to detect early obstetric complications (malposition, placenta praevia, twins, preeclampsia, infections, anemia) and identify complicated deliveries that should not be carried out in rural areas lacking of medical supervision. One thousand pregnant women have been attended using this kit, and results show that neonatal mortality has been reduced a 65% and maternal mortality has disappeared in the sample. The cost of the kit is around US $5500 and it allows attending an average of 400 pregnant women per year. A business model has been designed to scale the project, estimating a cost of $25 per pregnant woman. Now, with funding from AECID, USAID, and IDB, the Project is trying to increase the sample to 10,000 pregnant women only in Guatemala.

## Conflict of Interest Statement

The authors declare that the research was conducted in the absence of any commercial or financial relationships that could be construed as a potential conflict of interest.
